# Resistance Mechanisms of Rhizospheric *Bacillus* and *Pseudomonas* Strains Against Heavy Metal Contamination (Cu, Cr and Cd) and Their Antifungal Properties

**DOI:** 10.3390/microorganisms14030644

**Published:** 2026-03-12

**Authors:** Slimane Mokrani, Zahira Benouguef, Karim Houali, Leila Bensidhoum, Assia Derguini, Nasir A. Ibrahim, Nosiba S. Basher, El-hafid Nabti

**Affiliations:** 1 Laboratory of Research on Biological Systems and Geomantic (L.R.S.B.G.), Department of Biology, Mustapha Stambouli University, P.O. Box 305, Mascara 29000, Algeria; slimane.mokrani@univ-mascara.dz (S.M.); zahirabenouguef17@gmail.com (Z.B.); 2 Laboratory of Analytical Biochemistry and Biotechnology, Department of Microbiology and Biochemistry, Mouloud Mammeri University, Tizi Ouzou 15000, Algeria; karim.houali@ummto.dz; 3 Laboratoire de Maitrise Des Energies Renouvelables, Université de Bejaia, Bejaia 06000, Algeria; leila.bensidhoum@univ-bejaia.dz (L.B.); el-hafid.nabti@univ-bejaia.dz (E.-h.N.); 4 Microbial Ecology Laboratory, Department of Microbiology, Abderrahmane Mira University, Bejaïa 06000, Algeria; assia.derguini@univ-bejaia.dz; 5 Department of Biology, College of Science, Imam Mohammad Ibn Saud Islamic University, Riyadh 11623, Saudi Arabia; naabdalneim@imamu.edu.sa

**Keywords:** antifungal activities, *Bacillus*, *Pseudomonas*, heavy metals, resistance mechanisms

## Abstract

Environmental pollution caused by persistent chemical compounds, particularly heavy metals, poses a significant global challenge. Current strategies focus on eco-friendly and sustainable approaches, such as the application of microorganisms, to mitigate this issue. In this study, four strains of *Bacillus* and *Pseudomonas* were phylogenetically identified and assessed for their resistance to three heavy metals: copper (Cu), chromium (Cr), and cadmium (Cd) up to 500 µg/mL. Various tolerance mechanisms related to heavy metal resistance were elucidated, including salinity tolerance, antibiotic resistance, production of exopolysaccharides (EPS), and biosurfactant synthesis. The antifungal activities of these strains were evaluated against the fungal isolates *Fusarium oxysporum fs. phaseoli* (Fop) and *Stemphylium botryosum* (St-bt) using dual culture assays. Phylogenetic analysis revealed that three strains belong to the genus *Bacillus*, while one strain is classified under *Pseudomonas*. Additionally, these strains exhibited diverse mechanisms for heavy metal tolerance, including salinity tolerance (up to 600 mM), multi-antibiotic resistance (to imipenem, ampicillin, and sodium fusidate), and the production of viscous, slimy colonies indicative of EPS synthesis. Biosurfactant production led to a significant reduction in surface tension, ranging from 10.51 ± 3.87% to 82.89 ± 5.01%. The antifungal assays demonstrated that the strains effectively inhibited the mycelial growth of the fungal isolates, with inhibition percentages varying from 0% to 83.34 ± 2.22%. The strains characterized in this study exhibit considerable potential for application in the bioremediation of metal-contaminated soils and as biocontrol agents.

## 1. Introduction

Human activities have profoundly altered both the biotic and abiotic components of ecosystems. Environmental pollution, defined as the release of toxic materials into air, water, and soil, poses a major threat to all living organisms and ecosystem functioning. Even trace amounts of contaminants, whether in liquid, solid, or gaseous forms, can adversely affect ecosystem health. Large-scale pollution emerged with the Industrial Revolution and has since caused severe degradation of environmental quality and human health [1]. Although pollution is now recognized as a global health concern, its impacts are particularly acute in developing countries, where high pollution levels result from poverty, insufficient investment in clean technologies, and weak environmental regulations.

The relationships between environmental contamination and health outcomes are complex and often inadequately characterized. Recent international initiatives have aimed to quantify the global disease burden attributable to environmental pollution, particularly in terms of disability-adjusted life years (DALYs) and mortality. Pollution is estimated to account for 8–9% of the total global disease burden, with significantly higher proportions observed in developing nations [2].

Critical situations are driven by increasing food demand, intensive agriculture, rapid industrialization, deforestation, urbanization, population growth, unsustainable resource exploitation, and excessive consumption across various sectors. Industrial activities are major sources of toxic compounds that contaminate land, air, and water, persisting in the environment for extended periods. Concurrently, agricultural practices that rely on fertilizers, pesticides, and inadequately treated effluents significantly contribute to soil and water contamination [3].

Among the various forms of pollution, heavy metal contamination presents a particularly serious ecological challenge and frequently occurs in conjunction with other pollutants. Activities such as fossil fuel extraction, combustion, and refining release substantial amounts of metals into the environment [4]. Human actions have profoundly modified both regional and global biogeochemical cycles of many trace elements, resulting in widespread contamination of freshwater resources and the human food chain with hazardous metals [5]. Heavy metals are among the most pressing environmental pollutants due to their non-biodegradable nature, ability to accumulate along food chains, and association with a broad spectrum of adverse health effects in humans and other organisms [6].

A variety of physical, chemical, and biological methods are employed to manage heavy metal contamination. Conventional techniques such as ion exchange, adsorption, and reverse osmosis are considered effective and relatively safe for the environment. However, they are often costly, may generate secondary waste, and are unable to remove all chemical forms of heavy metals completely, thereby highlighting the need for more specialized and sustainable remediation strategies [7]. In this context, bioremediation has emerged as a promising alternative, exploiting the metabolic capabilities of specific microorganisms and plants to immobilize, transform, or remove heavy metals from contaminated soils and waters, offering an environmentally sound and cost-effective solution [8].

Plant-beneficial microorganisms (PBM), particularly plant growth-promoting bacteria (PGPB), play a crucial role at the plant–soil interface. They form associations with plant roots that can alleviate heavy metal toxicity, enhance plant tolerance to metals and other environmental stressors, and modulate the bioavailability of metals in the rhizosphere [9]. Under conditions of severe metal contamination, many bacterial taxa have evolved diverse resistance mechanisms, enabling them to metabolize, sequester, or transform heavy metals into less toxic forms, resulting in the emergence of multiple heavy metal-resistant bacterial species [10]. Microorganisms belonging to the genera *Bacillus*, *Enterobacter*, *Pseudomonas*, *Aspergillus*, and *Penicillium* have garnered particular interest due to their significant potential for bioremediation of metal-contaminated environments [11]. Beyond their roles in metal detoxification, bacterial agents can also suppress plant pathogens, serving as safer alternatives to conventional synthetic pesticides.

The genera *Bacillus* and *Pseudomonas* are among the most widely studied and applied biocontrol agents because of their prominent plant growth-promoting (PGP) traits [12]. Recent studies have indicated that specific strains within these genera can provide an environmentally friendly and integrated plant health management system by stimulating systemic resistance against both biotic and abiotic stresses [13]. Furthermore, certain microbes naturally inhabiting heavy metal-polluted soils exhibit high levels of metal tolerance, thereby contributing simultaneously to the reduction in metal toxicity and the enhancement of plant performance in contaminated environments.

Numerous bacteria utilize distinct survival strategies, such as biosorption and the secretion of metal-chelating molecules, to thrive in environments contaminated with heavy metals [14]. Common tolerance mechanisms include the conversion of metals into more soluble and bioavailable forms, bioaccumulation within microbial cells, the production of extracellular polymeric substances (EPS), and the synthesis of siderophores, among others [15]. In soils with high concentrations of heavy metals, bacteria also activate several plant-beneficial traits, notably the production of 1-aminocyclopropane-1-carboxylic acid (ACC) deaminase, synthesis of phytohormones, and formation of indole-3-acetic acid (IAA), thereby supporting plant growth under metal stress [16].

In addition, Nnaji et al. [17] highlighted the intricate relationships between bacterial communities and their capacity to withstand toxic metals, demonstrating how heavy metal contamination reshapes microbial community structure and function while selecting for specific metal-tolerant populations. These evolutionary adaptations—including biofilm formation, horizontal gene transfer (HGT), and various genetic modifications—underscore the remarkable resilience of bacteria to the environmental constraints imposed by heavy metals.

Tolerance to heavy metals (HMs) in microorganisms can arise from both chromosomal and plasmid-borne determinants. Proteins such as MerT and MerA, encoded by the mer operon, along with the products of the *czcCBA*, *ArsR*, *ArsA*, *ArsD*, *ArsB*, and *ArsC* genes, play key roles in metal detoxification within bacterial cells [18]. Many bacteria have developed multiple metal-resistance genes in response to chronic exposure to toxic elements, including arsenic, cadmium, chromium, copper, lead, and mercury, thereby enhancing their adaptive capacity to contaminated environments. These resistance determinants can be harnessed for the bioremediation of metal-polluted sites.

Several operon-clustered metal-resistance genes involved in cadmium, chromium, copper, lead, mercury, and nickel resistance and detoxification have been identified in bacterial systems, including *cadB*, *chrA*, *copAB*, *pbrA*, *merA*, and *NiCoT* [19]. These systems underpin diverse bacterial metal tolerance mechanisms—such as enzymatic detoxification, active efflux pumps, and biofilm formation—that collectively enable bacterial populations to adapt to and persist in polluted habitats. Both horizontal gene transfer and spontaneous or induced gene mutations further enhance these adaptive traits, allowing bacterial species not only to withstand environmental stress but also to contribute to nutrient cycling and the degradation of organic matter in heavy metal-contaminated ecosystems [17].

Assessing bacterial resistance usually involves the isolation, screening, and characterization of microbial strains. Numerous isolates, particularly species belonging to the genera *Bacillus* and *Pseudomonas*, have demonstrated strong resistance to metals such as Pb, Hg, and Zn, underscoring their promising potential for the bioremediation of heavy metal-contaminated environments [20]. In this context, the present study aims to evaluate the resistance capacities of selected *Bacillus* and *Pseudomonas* strains to the heavy metals Cu, Cr, and Cd, while also examining associated functional traits, including salinity tolerance, antibiotic resistance, exopolysaccharide (EPS) synthesis, biosurfactant production, and antifungal activities.

## 2. Materials and Methods

### 2.1. Bacterial Strains and Isolation Sources

This study utilized four bacterial strains originally isolated in 2010 from soil samples collected in the Sidi Belabbes and Mascara departments of western Algeria. The samples were obtained from both bulk and rhizospheric soils associated with *Phaseolus vulgaris* L. and *Allium cepa* L. Following their initial isolation and phylogenetic characterization between 2010 and 2015, the strains were preserved at −20 °C in Tryptic Soy Broth (TSB) supplemented with 25% (*v/v*) glycerol [21]. All subsequent physiological experiments, including assessments of heavy metal tolerance, salinity tolerance, antibiotic resistance, exopolysaccharide (EPS) production, biosurfactant synthesis, and antifungal activity, were conducted in 2024. Detailed information on the isolation sources and geographical coordinates is provided in Table 1.

### 2.2. Phylogenetic Identification and Analysis

Genomic DNA was extracted from pure bacterial cultures using a phenol/chloroform/isoamyl alcohol precipitation method, following the procedure described by William et al. [22]. The 16S rRNA gene was amplified by PCR using the universal primers 16SF (5′-AGA GTT TGA TGA TCC TGG CTC AG-3′) and 16SR (5′-CTA CGG CTA CCT TGT TAC GA-3′). The resulting sequences were compared against the GenBank database using the BLASTn algorithm (version 2.14.0+) [23]. Multiple sequence alignment was performed with ClustalW, and evolutionary distances were computed using the Kimura two-parameter (K2P) model [24]. A phylogenetic tree was constructed using the Neighbor-Joining method within MEGA7 software (MEGA version 7.0.26-1) [25] to determine the phylogenetic affiliation of the isolates.

### 2.3. Evaluation of Heavy Metal Tolerance

Heavy metal tolerance was assessed using a turbidimetric method adapted from Deshpande et al. [26]. Overnight bacterial cultures were adjusted to 10^6^ CFU/mL in sterile saline. Aliquots (1 mL) of this suspension were used to inoculate test tubes containing 9 mL of Luria-Bertani (LB) broth supplemented with filter-sterilized solutions of copper (II) sulfate (CuSO_4_), potassium dichromate (K_2_Cr_2_O_7_), or cadmium sulfate (CdSO_4_). The final metal concentrations tested were 50, 100, 200, 400, and 500 µg/mL. For each metal concentration, an abiotic control tube (uninoculated LB broth with the respective metal concentration) was included and incubated under identical conditions. All tubes were incubated at 30 °C for 24 h with shaking at 150 rpm. Bacterial growth was quantified by measuring optical density at 625 nm (OD_625_). Prior to each measurement, the spectrophotometer was blanked using the corresponding abiotic control to correct for any potential interference from metal precipitates or medium turbidity. For each strain and metal concentration, three independent biological replicates were performed, each with three technical replicates (*n* = 9 total measurements per condition). Results were expressed as mean OD_625_ ± standard deviation (SD).

### 2.4. Characterization of Mechanisms Potentially Linked to Metal Tolerance

#### 2.4.1. Salinity Tolerance

Salinity tolerance was evaluated by growing strains in LB broth supplemented with 0, 200, 400, or 600 mM NaCl, following a modified protocol from Praveen Kumar et al. [27]. Tubes containing 9 mL of the respective media were inoculated with 1 mL of bacterial suspension (10^6^ CFU/mL) and incubated at 30 °C for 24 h. Growth was assessed by measuring OD_625_, using uninoculated medium as a blank. Strains achieving an OD_625_ ≥ 0.100 were considered tolerant to the tested salt concentration. Three independent biological replicates were performed for each strain and NaCl concentration.

#### 2.4.2. Antibiotic Resistance Profiling

Antibiotic susceptibility was determined using the disk diffusion method on Mueller-Hinton (MH) agar, according to the guidelines of the Antibiogram Committee of the French Society for Microbiology (CA-SFM) [28,29]. Bacterial suspensions were adjusted to a 0.5 McFarland standard and uniformly swabbed onto MH agar plates. Disks containing the following antibiotics were applied: amikacin (AK, 30 µg), imipenem (IMP, 10 µg), ampicillin (AM, 10 µg), gentamicin (GEN, 10 µg), and sodium fusidate (FC, 10 µg). This panel was selected to cover different mechanisms of action (cell wall synthesis inhibitors and protein synthesis inhibitors) and to allow investigation of potential co-selection with metal tolerance, as frequently reported in bacteria from polluted environments [17,30]. After incubation at 30 °C for 24 h, the diameters of the inhibition zones were measured. Strains were classified as Susceptible (S) or Resistant (R) according to CA-SFM breakpoints [31]. For *Bacillus* spp., where specific CA-SFM breakpoints were unavailable for some antibiotics, staphylococcal breakpoints were applied as references [32]. For *Pseudomonas* spp., resistance was defined as an inhibition zone diameter ≤ 1 mm [32,33].

#### 2.4.3. Exopolysaccharide (EPS) Production

Qualitative EPS production was assessed by streaking strains onto MSE agar medium supplemented with 10% sucrose [34]. Plates were incubated at 37 °C for 24–48 h. EPS production was inferred from the appearance of viscous, mucoid, and slimy colonies. The assay was performed in triplicate for each strain to ensure reproducibility.

#### 2.4.4. Biosurfactant Production Assay

Biosurfactant production was evaluated using a method adapted from Morikawa et al. [35]. Single colonies were inoculated into 50 mL of TSB supplemented with 2% (*v/v*) olive oil in Erlenmeyer flasks. Cultures were incubated at 37 °C for 72 h with manual agitation every 20 h. The ability of the culture supernatant to reduce surface tension was expressed as the percentage of surface tension reduction (STR), calculated using the following equation [36]:

STR=(ym−yc)/ym×100

where:

STR = surface tension reduction (%), ym = surface tension of the prepared medium solution, and yc = surface tension of the culture medium. All experiments were conducted in triplicate (*n* = 3), and results were expressed as mean ± standard deviation.

#### 2.4.5. Antifungal Activity Assays

##### Fungal Pathogens

The phytopathogenic fungi *Fusarium oxysporum* f. sp. *phaseoli* (Fop) [37] and *Stemphylium botryosum* (St-bt) [38], previously isolated from diseased *Phaseolus vulgaris* L. plants, were used as target organisms.

##### Dual Culture Assay

Antifungal activity was evaluated using a dual culture technique adapted from Landa et al. [39]. A 6 mm agar plug of a 7-day-old fungal culture was placed at the center of a Petri dish containing Potato Dextrose Agar (PDA). Four 6 mm agar plugs of a 24 h bacterial culture were then placed equidistantly around the fungal plug at a distance of 4 cm. Plates were incubated at 25 °C for 5–7 days. Fungal growth inhibition was quantified by calculating the percentage of inhibition (PI) [40]:

PI=(r control−r test)/r control×100

where r control: maximum radial distance of the fungus growth; r test: radial distance growth on a line in the direction of the antagonist. The control consisted of phytopathogenic fungus transplanted alone in the center of Petri plate.

### 2.5. Statistical Analysis

The resistance of bacterial strains to various abiotic and biotic stresses—including heavy metals, salinity, biosurfactant production, and antifungal activity—was statistically evaluated. All experiments were performed in triplicate (*n* = 3), and data were expressed as mean ± standard deviation (SD). Prior to analysis, normality of the data was verified using the Shapiro–Wilk test. A one-way analysis of variance (ANOVA) was then conducted using GraphPad Prism (version 9.0 for Windows, GraphPad Software, San Diego, CA, USA), followed by Tukey’s multiple comparison test to identify significant differences between treatment groups. Differences were considered statistically significant at *p* < 0.05.

## 3. Results

### 3.1. Phylogenetic Identification

**Figure 1:**
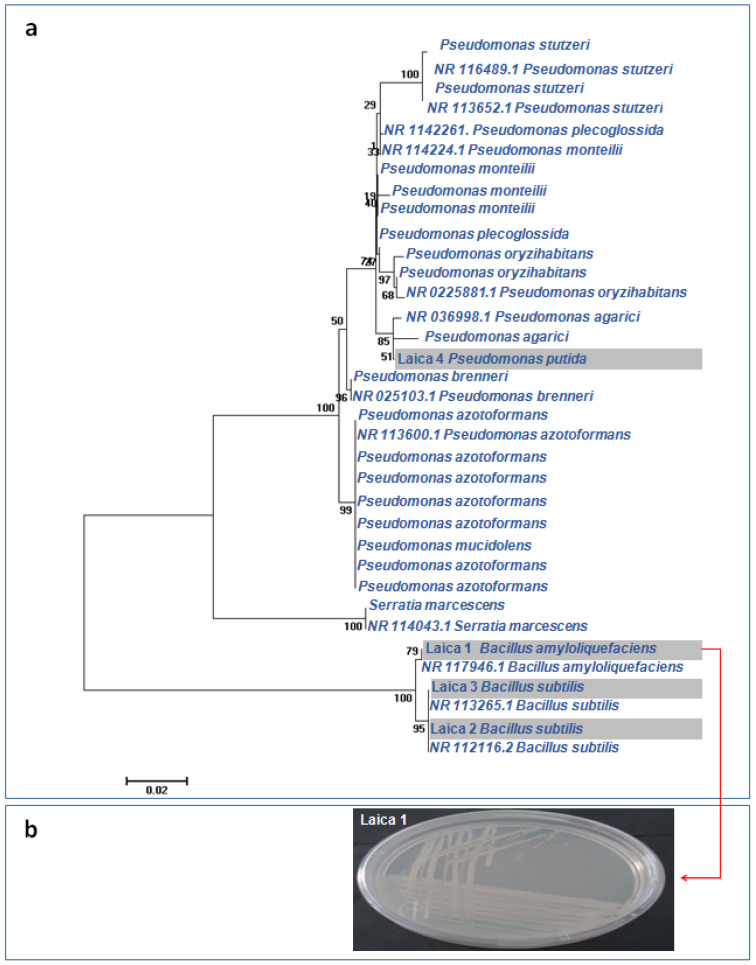
Phylogenetic identification of the bacterial strains. (**a**) Phylogenetic tree of *Bacillus* and *Pseudomonas* strains (bar indicates 0.02% divergence). (**b**) macroscopic aspect of the screened strain Laica 1 on TSA agar.

The phylogenetic tree constructed from 16S rRNA gene sequences clarified the relationships among the four bacterial strains, which belonged to the genera *Bacillus* (three strains) and *Pseudomonas* (one strain) (Figure 1a). Strain Laica 1 (*Bacillus amyloliquefaciens*) formed a distinct clade together with the reference strain *Bacillus amyloliquefaciens* NR 117946.1 (GenBank accession). The other two *Bacillus* strains, Laica 2 (*Bacillus subtilis*) and Laica 3 (*Bacillus subtilis*), clustered in a separate clade that also included the reference strains *Bacillus subtilis* NR 113265.1 and *Bacillus subtilis* NR 112116.2. Strain Laica 4 (*Pseudomonas putida*) grouped within a clade comprising closely related *Pseudomonas* strains, including *Pseudomonas agarici and Pseudomonas agarici* NR 036998.1.

Figure 1b presents the macroscopic morphology of strain Laica 1 grown on TSA agar, as described in the following section.

### 3.2. Heavy Metals Tolerance

**Figure 2:**
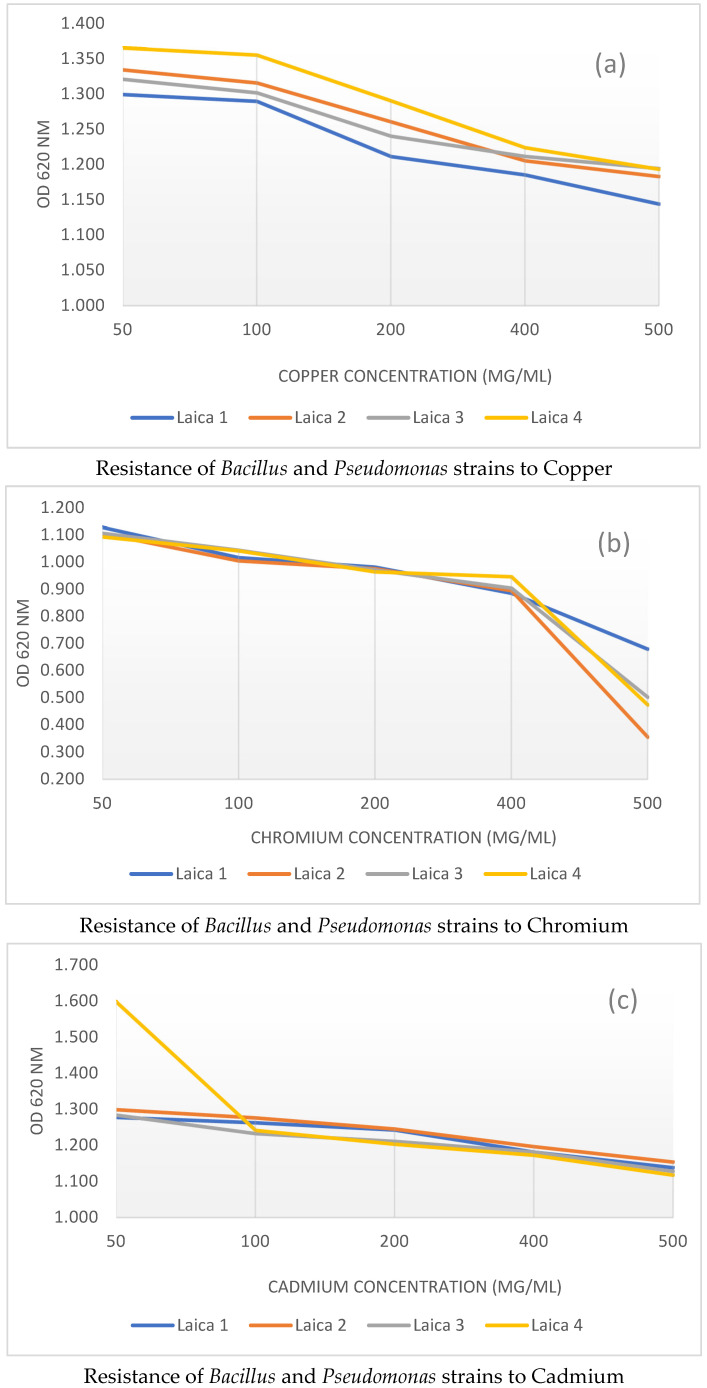
Resistance of *Bacillus* and *Pseudomonas* strains to Heavy metal (Cu, Cr, and Cd).

As shown in Figure 2, the assessment of heavy metal tolerance in *Bacillus* and *Pseudomonas* strains revealed marked variability in their resistance to the three tested metals: copper (Cu) (Figure 2a), chromium (Cr) (Figure 2b), and cadmium (Cd) (Figure 2c) across the different concentrations. The following section details these experimental findings and underscores the notable resilience of several strains to heavy metal toxicity.

#### 3.2.1. Copper Sulfate Tolerance

The growth response of the bacterial strains to copper sulfate was systematically evaluated by monitoring optical density at increasing Cu concentrations (Figure 2a). A progressive decline in OD was observed as the copper sulfate concentration increased. At the lowest concentrations tested (50 and 100 μg/mL), all strains exhibited high levels of resistance, with OD values ranging from 1.299 ± 0.002 to 1.365 ± 0.003 at 50 μg/mL and from 1.290 ± 0.002 to 1.355 ± 0.005 at 100 μg/mL, indicating that the strains can still grow efficiently under low copper stress. In contrast, when the Cu concentration exceeded 100 μg/mL, the inhibitory effect on bacterial growth became more pronounced, and at 500 μg/mL, OD values dropped to between 1.144 ± 0.003 and 1.194 ± 0.004. This marked reduction in growth at the highest concentration suggests that copper toxicity reaches a threshold beyond which these strains are significantly affected.

Across the concentration range tested (50–500 µg/mL), strain Laica 4 exhibited the highest level of copper resistance, followed by strains Laica 2 and Laica 3, respectively. Strain Laica 1 was the least resistant to copper among the four tested strains.

#### 3.2.2. Potassium Dichromate Tolerance

The resistance of the strains to potassium dichromate was also evaluated, and the results indicated high tolerance at concentrations between 50 and 400 μg/mL (Figure 2b). At these levels, only slight reductions in optical density were observed, with OD values ranging from 1.093 ± 0.001 to 1.128 ± 0.002 at 50 μg/mL and from 0.885 ± 0.008 to 0.946 ± 0.004 at 400 μg/mL. These findings suggest that the strains possess effective detoxification mechanisms that allow them to withstand moderate potassium dichromate stress. In sharp contrast, exposure to 500 μg/mL of potassium dichromate caused a pronounced decrease in OD, with values dropping to between 0.354 ± 0.004 and 0.679 ± 0.009, highlighting a toxicity threshold beyond which the strains are no longer able to sustain normal growth.

At chromium concentrations between 50 and 400 µg/mL, the four strains displayed comparable levels of resistance, as reflected by the very similar growth curves. Above 500 µg/mL, a marked reduction in resistance was observed in all strains. Strain Laica 1 was the most resistant, followed by Laica 3 and Laica 4, which exhibited similar resistance profiles, whereas Laica 2 was the least resistant to chromium.

#### 3.2.3. Cadmium Sulfate Tolerance

For cadmium sulfate, all strains exhibited robust resistance at 50 μg/mL, with optical densities ranging from 1.284 ± 0.004 to 1.598 ± 0.573. Notably, strain Laica 4 showed the highest resistance, with an OD of 1.598 ± 0.573 at 50 μg/mL, suggesting that it may possess specific adaptations conferring enhanced tolerance to cadmium (Figure 2c)

As the cadmium sulfate concentration increased above 100 μg/mL, a gradual decline in optical density was observed, with minimum values at 500 μg/mL ranging from 1.117 ± 0.003 to 1.154 ± 0.004. This trend reflects reduced bacterial viability at higher cadmium levels and confirms that cadmium imposes a substantial stress on these strains.

At a cadmium concentration of 50 µg/mL, strain Laica 4 was the most resistant, followed by Laica 3 and Laica 1, respectively. Between 50 and 400 µg/mL, the four strains showed very similar growth curves, indicating comparable levels of resistance, whereas at 500 µg/mL Laica 2 was the most resistant and Laica 4 the least resistant to cadmium.

#### 3.2.4. Summary of Heavy Metal Resistance

In summary, at the maximum concentration of 500 μg/mL, strain Laica 1 (*Bacillus amyloliquefaciens*) exhibited the highest overall resistance to copper and chromium, with OD values of 1.144 ± 0.003 for copper and 0.679 ± 0.009 for chromium. For cadmium, the OD of Laica 4 at 50 μg/mL (Cd) was markedly higher than that of the other strains, and at 500 μg/mL the OD values of all strains converged to similar levels, indicating that Laica 4 (*Pseudomonas* putida) can be considered the most cadmium-resistant strain. These findings underscore the strong potential of Laica 1 for bioremediation applications, as its tolerance to elevated copper and chromium concentrations suggests the presence of efficient detoxification mechanisms, while the variability in metal tolerance among strains highlights the need for further studies on the underlying resistance pathways that could be exploited in environmental strategies to mitigate heavy metal pollution.

It is important to emphasize that these results primarily reflect growth tolerance under controlled laboratory conditions rather than definitive metal resistance. Metal toxicity and microbial responses are strongly influenced by metal bioavailability and chemical speciation, rather than by the total metal concentration alone.

Statistical data analysis revealed that resistances of *Bacillus* and *Pseudomonas* strains to Cu, Cr, and Cd were significant (Table 2).

### 3.3. Mechanisms Related to Heavy Metals Tolerance

#### 3.3.1. Salinity Tolerance

**Figure 3:**
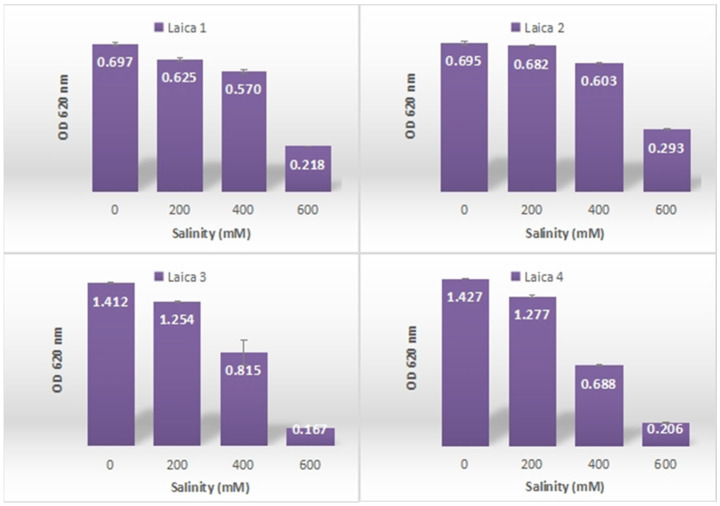
Resistance of *Bacillus* and *Pseudomonas* strains to salinity.

The assessment of salt tolerance among the bacterial strains revealed clear variability in growth, indicative of differing levels of NaCl resistance (Figure 3). At 200 and 400 mM NaCl, all strains showed high to moderate tolerance, with OD values ranging from 0.625 ± 0.006 for Laica 1 to 1.277 ± 0.006 for Laica 4, confirming that they can still grow under moderate salinity stress.

At 600 mM NaCl, a pronounced reduction in OD was observed for all strains, with minimum and maximum values of 0.167 ± 0.004 for Laica 3 and 0.293 ± 0.003 for Laica 2, respectively. Under these conditions, Laica 2 was the most salt-tolerant strain, followed by Laica 1 (OD 0.218 ± 0.002), highlighting distinct differences in salinity tolerance and suggesting that specific physiological or molecular mechanisms may enable certain strains to better withstand high salt concentrations

Statistical analysis indicated that the resistance of *Bacillus* and *Pseudomonas* strains to salinity was significant (Table 3). This suggests that the variations in salinity tolerance among the strains are not due to random chance, but rather reflect inherent differences in their physiological responses to saline conditions.

#### 3.3.2. Antibiotic Resistance

Statistical analysis showed that the antibiotic resistance patterns of the *Bacillus* and *Pseudomonas* strains were significantly different (Table 4). This indicates that the observed variations in antibiotic tolerance are not due to random variation but instead reflect intrinsic differences in their physiological and biochemical responses to antibiotic exposure, likely influenced by genetic determinants, specific metabolic pathways, and distinct resistance mechanisms that help these strains withstand antibiotic pressure.

#### 3.3.3. Synthesis of Exopolysaccharides (EPS)

The assessment of exopolysaccharide (EPS) production by the *Bacillus* and *Pseudomonas* strains showed that Laica 2 and Laica 4 were effective EPS producers, as evidenced by their viscous, sticky colonies typical of active EPS synthesis (Table 5). In contrast, Laica 1 and Laica 3 did not display any visible EPS production under the tested conditions.

**Figure 4:**
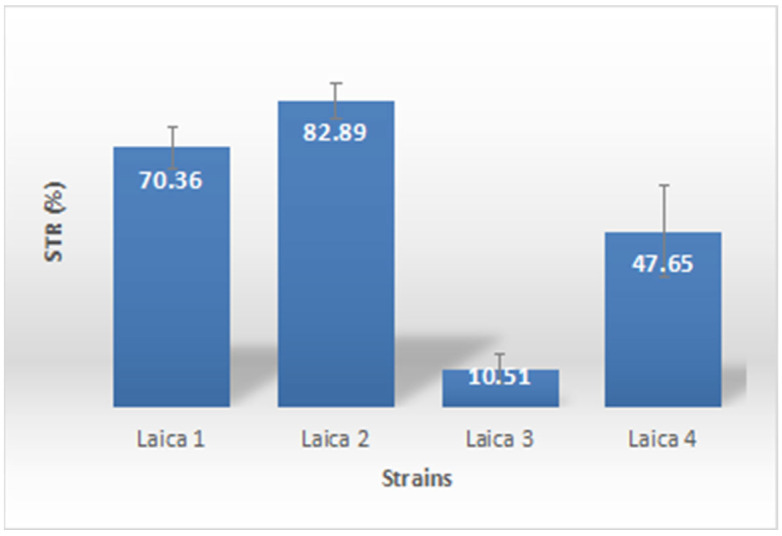
Production of Biosurfactants by *Bacillus* and *Pseudomonas* strains (STR: Surface Tension Reduction).

The current findings confirmed biosurfactant production in all *Bacillus* and *Pseudomonas* strains, as demonstrated by surface tension measurements in TSB broth supplemented with 2% olive oil (Figure 4). Surface tension reduction varied widely among strains, from 10.51 ± 3.87% to 82.89 ± 5.01%, indicating differential biosurfactant synthesis capacity. The highest reduction was recorded for strain Laica 2 (82.89 ± 5.01%), followed by Laica 1 (70.36 ± 5.54%), highlighting these strains as particularly efficient biosurfactant producers with promising potential for biotechnological applications such as bioremediation and microbial enhanced oil recovery.

Statistical analysis of the biosurfactant production results, conducted using one-way ANOVA followed by Tukey’s multiple comparison tests, revealed significant differences among most treatments (Table 6).

### 3.4. Antifungal Activities

**Figure 5:**
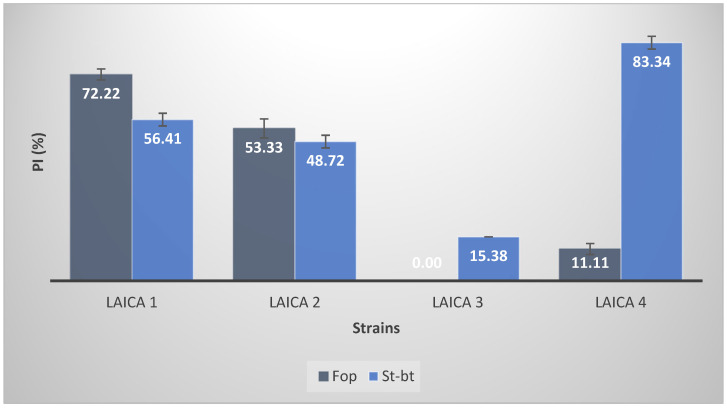
Antifungal Activities of *Bacillus* and *Pseudomonas* Strains Against Phytopathogenic Fungi. (PI: Percentage Inhibition; Fop: *Fusarium oxysporum* f. sp. *phaseoli*; St-bt: *Stemphylium botryosum*).

The antifungal activity of the *Bacillus* and *Pseudomonas* strains showed clear variability in the percentage of mycelial growth inhibition against the fungal isolates St-bt and Fop (Figure 5). Against St-bt, strain Laica 4 displayed the strongest effect, with 83.34 ± 2.22% inhibition, followed by Laica 1 with 56.41 ± 2.22% inhibition.

In contrast, for the isolate Fop, Laica 1 achieved 72.22 ± 1.92% inhibition, whereas Laica 2 produced a lower but still substantial inhibition of 53.33 ± 3.34%. These findings indicate that some strains, particularly Laica 4 and Laica 1, possess marked antifungal properties that could be exploited for biocontrol in agriculture and plant health management.

Statistical analysis of the antifungal activity results, conducted using one-way ANOVA followed by Tukey’s multiple comparison tests, revealed significant differences among all treatments (Table 7).

## 4. Discussion

Understanding how microorganisms withstand harmful environmental pollutants, particularly heavy metals, is crucial both for elucidating resistance mechanisms and for identifying strains suitable for the bioremediation of contaminated sites. In the present study, the *Bacillus* and *Pseudomonas* strains tolerated copper (Cu), chromium (Cr), and cadmium (Cd) at concentrations up to 500 µg/mL, which is consistent with their origin from metal-contaminated soils. Heavy metal resistance in bacteria can result from redox transformations that modify metal speciation [41] and from metabolism-dependent uptake mechanisms that enable intracellular sequestration [42,43]. Moreover, prolonged exposure to elevated metal concentrations in soils may enhance microbial tolerance and adaptive resistance while simultaneously altering key ecological processes such as nitrogen cycling and organic matter decomposition [44,45]. Sustained metal exposure can therefore increase the overall resilience of microbial communities [46].

A detailed understanding of microbial tolerance at high metal concentrations is essential for selecting suitable species for bioremediation and biotransformation applications [47]. The link between bacterial resistance and bioremediation is fundamental to addressing heavy metal pollution. Resistant bacteria can persist in contaminated environments and transform toxic metals by reducing or methylating them into less harmful forms, thereby directly contributing to detoxification. In addition, plasmid-encoded resistance genes within microbial communities can improve remediation efficiency by promoting adaptation to, and neutralization of, a wide range of metals [11].

Speciation refers to the identification and quantification of the different chemical species, forms, or phases in which an element occurs [48]. It is also the field that determines a metal’s chemical form and oxidation state, which largely control its behavior in the environment [49]. Metal and semi-metal speciation plays a central role in determining their biological effects, as different elements, ions, and compounds can display markedly different toxicity profiles in exposed organisms [50]. To correctly interpret adverse biological effects, knowledge of metal speciation is indispensable. Generic descriptors such as “heavy” or “toxic” do not adequately reflect the biological properties of individual elements, which depend on their precise chemical structures [50]. Speciation in biotic and abiotic media strongly influences metal bioavailability, bioaccumulation, and toxicity for receptor species [49]. In general, ionic salts are the most bioavailable and reactive forms, whereas the elemental metallic form tends to be comparatively inert [50]. In this study, the *Bacillus* and *Pseudomonas* strains also exhibited salt tolerance, growing at NaCl concentrations up to 600 mM.

Microorganisms typically cope with high-salinity environments through two complementary strategies: the synthesis or uptake of compatible organic solutes and the accumulation of K^+^ and other inorganic ions to counterbalance osmotic stress [51,52]. These mechanisms help maintain cellular homeostasis and support normal physiological functions under elevated osmotic pressure [53]. Halophilic and halotolerant microorganisms are especially promising for bioremediation and biotransformation because of their distinctive physiological adaptations. For example, many halophilic bacteria require high concentrations of specific anions and cations for optimal growth [54]. As a result, they can tolerate, or even depend on, elements that are toxic to many other microorganisms.

Furthermore, some heavy metal-resistant halophilic bacteria can serve as useful bioindicators in saline, metal-contaminated environments, underscoring the importance of studying their resistance and ecology [55].

Antibiotic susceptibility testing revealed that the *Bacillus* and *Pseudomonas* strains were multidrug-resistant to several antibiotics, including imipenem, ampicillin, and sodium fusidate. Many *Pseudomonas* isolates are known to display variable tolerance to both antibiotics and heavy metals, reflecting a broad adaptive capacity. Microbial resistance to heavy metals relies on multiple detoxification strategies, including complexation by exopolysaccharides, binding to cell envelopes, enzymatic metal reduction, and active efflux systems [56]. Some of these resistance determinants are plasmid-borne, favoring horizontal transfer to other bacterial cells and thereby promoting the spread of metal resistance within microbial communities [57]. Fu et al. [58] showed that the rapid intensification of livestock and poultry production has increased the input of heavy metals and antibiotics into soils through feed additives, yet the relationships among environmental parameters, microbial communities, and antibiotic resistance genes (ARGs) remain only partially understood. Using cadmium (Cd) and sulfadiazine (SD) as model contaminants, they investigated the interactions among microbes, ARGs, mobile genetic elements (MGEs), and environmental factors, highlighting the complexity of co-selection processes in contaminated ecosystems [58].

In the present work, exopolysaccharide (EPS) production was detected in strain Laica 2 (*Bacillus subtilis*) and strain Laica 4 (*Pseudomonas putida*). It must be stressed that EPS production was inferred solely from colony morphology, which provides only preliminary evidence; more advanced analyses, such as quantitative EPS measurements and detailed chemical characterization, are needed to confirm and better describe EPS production. EPS is a naturally occurring extracellular material located at the surface of many bacterial cells, where its structure can promote the sequestration of metal ions and limit their access to the cell envelope [59]. For instance, *Pseudomonas* sp. PFAB4 produces EPS in response to Ag^+^ exposure, forming an extracellular barrier that restricts heavy metal entry into cells [60]. Lau et al. [61] monitored Cu(II) adsorption using a dye displacement method after partial purification of capsular EPS from *Pseudomonas* sp. CU-1 and found that the Cu(II) sorption capacity of EPS (0.32 mmol·g^−1^) was only slightly lower than that of whole-cell pellets (0.33 mmol·g^−1^), indicating that EPS effectively shields cell surfaces from copper. Moreover, EPS from a resistant strain accumulated 1.2-fold more Cu (II) than EPS from a copper-sensitive strain, further supporting the role of EPS in metal sequestration and resistance [62]. In our study, EPS production appears to depend on species identity, genetic background, and isolation source, as illustrated by the EPS-producing strains *Bacillus subtilis* (Laica 2) and *Pseudomonas putida* (Laica 4). Numerous bacterial species, including these two, are known to synthesize exopolysaccharides, although both the quantity and the composition of EPS can vary markedly with species and growth conditions [63,64]. In *Bacillus subtilis*, EPS also acts as a signaling molecule that regulates its own synthesis; a dedicated tyrosine kinase complex, composed of the membrane component EpsA and the kinase component EpsB, is required for EPS production [65]. In *Pseudomonas putida* GAP-P45, exopolysaccharide synthesis under different abiotic stress conditions has been associated with soil aggregation, emphasizing the ecological importance of EPS in structuring the soil matrix [66]. The type and composition of EPS differ among bacterial strains [67], and environmental factors together with nutrient availability influence both the ecological niches from which EPS-producing bacteria can be isolated and the traits they express [68]. Biofilm formation by *Bacillus subtilis* is a well-characterized example: biofilms consist of chains of cells embedded in an extracellular matrix composed of EPS and the protein TasA.

EPS is synthesized by enzymes encoded by the epsA–O operon, whereas TasA is produced from the yqxM–sipW–tasA operon; both operons are repressed by SinR, and derepression is mediated by the antirepressor SinI, which binds SinR in a 1:1 ratio. Under conditions that promote derepression of the matrix operons, the cellular concentration of SinR nevertheless remains much higher than that of SinI, indicating a finely tuned regulatory balance that governs matrix and EPS production in *B. subtilis* biofilms [69]. Biosurfactants are microbial metabolites that enhance the solubility, bioavailability, and biodegradation of environmental contaminants [70]. Their amphiphilic nature and structural diversity allow them to reduce surface and interfacial tension and to promote micelle and microemulsion formation between otherwise immiscible phases, thereby increasing contaminant bioavailability and facilitating biodegradation [71].

In contrast to many organic pollutants, heavy metals are usually adsorbed onto soil particles as ions or charged ion pairs and enter the soil solution mainly through ion exchange or the formation of non-ionic complexes [72]. Overall, our assessments of EPS and biosurfactant production remain preliminary, and any mechanistic interpretation of their roles in metal sequestration and detoxification should be viewed with caution and confirmed by more detailed studies. Nonetheless, several mesocosm experiments have demonstrated that well-characterized, heavy metal-resistant bacterial strains offer a promising and environmentally friendly strategy for remediating contaminated sites. Maity et al. [73] isolated a multi-metal-resistant, Gram-positive, non-virulent *Bacillus* sp. GH-s29 strain from contaminated groundwater in Bhojpur district, Bihar, India, which formed biofilms capable of removing several metals, including arsenic, cadmium, and chromium, from both single- and multi-metal solutions, with maximum removal efficiencies for As(V), Cd(II), and Cr(VI) of 73.65%, 57.37%, and 61.62% (single-metal) and 48.92%, 28.7%, and 35.46% (multi-metal), respectively. In another study, the Zn (II) biosorption capacity of *Pseudomonas* sp. RY12 was evaluated in aqueous medium; at 28 °C (pH 6.5; initial Zn^2+^ concentration 200 mg/L), the strain reduced Zn^2+^ levels by up to 89% [74].

Changes in the expression of resistance genes have also been observed under heavy metal stress. Using semi-quantitative RT-PCR, one study examined gene expression patterns associated with metal resistance in several Gram-positive and Gram-negative bacteria highly resistant to Co^2+^ and Cd^2+^ and showed that these strains carried *mer*, *chr*, *czc*, and *ncc* genes implicated in metal resistance; however, Co^2+^ and Cd^2+^ exposure caused downregulation of *merA* and *chrB* in *Bordetella* sp., *Pseudomonas* sp., *Bacillus cereus*, *Bacillus subtilis*, and *Staphylococcus aureus* [75]. Khanna et al. [76] further demonstrated that metal-resistant plant growth-promoting rhizobacteria (PGPR) can enhance growth and photosynthetic pigment content in *Lycopersicon esculentum* under metal toxicity by reducing Cd uptake and downregulating metal transporter gene expression. Several studies have sought to link heavy metal resistance with associated traits such as antibiotic production, siderophore synthesis, and biofilm formation, as well as to identify the genes underlying these adaptive responses. The originality of the present work lies in its integrative perspective, which examines the relationship between heavy metal resistance and multiple associated mechanisms, including EPS and biosurfactant production, antibiotic tolerance, and salinity resistance.

This study is therefore distinctive in attempting to explain the heavy metal resistance of rhizospheric *Bacillus* and *Pseudomonas* strains through the combined contribution of these different traits. The strains characterized here may have realistic potential for field-scale application in the bioremediation of heavy metal-contaminated soils. In particular, strain Laica 1 (*Bacillus amyloliquefaciens*) exhibited the highest resistance to copper and chromium, whereas strain Laica 4 (*Pseudomonas putida*) showed the greatest resistance to cadmium. However, the study has several limitations. Heavy metal resistance was inferred solely from optical density measurements, which provide an indirect assessment of bacterial growth and do not distinguish between viable and non-viable cells. Similarly, associated mechanisms (EPS and biosurfactant production, antibiotic tolerance, salinity resistance) were evaluated with preliminary assays only. Consequently, the present findings require confirmation by more quantitative experiments and, crucially, by genetic and molecular investigations aimed at identifying the determinants of heavy metal resistance and associated traits and clarifying their roles at the molecular level.

The antifungal activities of the *Bacillus* and *Pseudomonas* strains resulted in mycelial growth inhibition ranging from 0 to 83.34 ± 2.22% against the fungal isolates St-bt and Fop. Because fungal infections are a major cause of postharvest losses in fruits and vegetables, there is a strong demand for safer and more environmentally friendly alternatives to synthetic fungicides, including the use of plant growth-promoting bacteria (PGPB) [77]. Biological control is considered one of the most promising strategies for disease management, especially in organic and protected vegetable production systems [78]. Morales-Cedeño et al. [77] evaluated the in vitro biocontrol potential of four well-characterized PGPB strains (*Bacillus toyonensis* COPE52, *Bacillus* sp. E25, *Bacillus thuringiensis* CR71, and *Pseudomonas fluorescens* UM270) against 19 postharvest fungal pathogens and showed that all strains significantly reduced disease incidence and improved fruit firmness. Among them, strain UM270 displayed outstanding biocontrol activity, reducing the incidence of *Alternaria alternata*, *Botrytis cinerea*, and *Fusarium brachygibbosum* on strawberry fruits by 60%, 55%, and 65%, respectively [77]. These beneficial effects were associated with the production of diffusible antifungal metabolites and volatile organic compounds (VOCs), including N,N-dimethyl-hexadecylamine, siderophores, auxins, fengycins, and 2,4-diacetylphloroglucinol, among others [77]. In our study, the *Bacillus* and *Pseudomonas* strains also produced biosurfactants, as indicated by their ability to reduce surface tension. However, biosurfactant production was inferred solely from surface tension measurements, providing only a preliminary estimate; more detailed analyses, including the identification of biosurfactant classes, are needed for definitive confirmation. The association between biosurfactant production and antifungal activity observed here is therefore particularly noteworthy. Strain Laica 2, which induced the greatest surface tension reduction (82.89 ± 5.01%), also showed strong antifungal activity (53.33 ± 3.34%) against Fop and moderate activity against St-bt. In contrast, Laica 3 exhibited the lowest biosurfactant levels (surface tension reduction 10.51 ± 3.87%) and consistently displayed the weakest antifungal effects against both fungal targets (Figure 5). This pattern suggests that biosurfactants may contribute directly to fungal growth inhibition, most likely through membrane-disrupting mechanisms. Biosurfactants produced by *Bacillus* species—particularly lipopeptides such as iturins, fengycins, and surfactins—are well-established antifungal agents [79,80]. These compounds insert into fungal membrane bilayers, forming ion channels or pores that compromise membrane integrity, ultimately causing leakage and cell death [81]. Iturins exhibit strong antifungal activity against a broad spectrum of phytopathogens, including *Fusarium* species, whereas fengycins show particular efficacy against filamentous fungi by inducing membrane permeabilization and apoptosis-like cell death [82]. Surfactins, although primarily hemolytic and antibacterial, can act synergistically with iturins and fengycins to enhance overall antifungal efficacy [83]. The variability in biosurfactant production among our strains likely reflects differences in their lipopeptide profiles. For instance, the high biosurfactant activity of Laica 2 may result from the simultaneous production of several lipopeptide classes, whereas the weak activity of Laica 3 could indicate low or absent synthesis of these compounds. In *Pseudomonas putida* Laica 4, which also produced biosurfactants and displayed the strongest antifungal activity against St-bt (83.34 ± 2.22%), the antifungal effects may be mediated by other types of biosurfactants, such as rhamnolipids or viscosin-like cyclic lipopeptides, which are known to disrupt fungal membranes and inhibit spore germination [84,85].

Although our data support a correlation between biosurfactant production and antifungal activity, definitive identification of the responsible compounds will require analytical characterization (e.g., LC-MS, MALDI-TOF) of the biosurfactants produced by each strain. Even so, the consistent observation that higher biosurfactant production coincides with stronger antifungal effects reinforces the hypothesis that biosurfactants contribute directly to the observed biocontrol activity.

This mechanistic insight strengthens the relevance of these strains for integrated pest management strategies, where biosurfactant-producing biocontrol agents can simultaneously suppress pathogens and improve the bioavailability of nutrients or metals [86]. Despite the limitations discussed above, the present study provides valuable insight into the heavy metal resistance mechanisms and associated traits of the isolated *Bacillus* and *Pseudomonas* strains and helps to place these findings in a broader ecological and applied context. The integrative approach adopted here—simultaneously examining heavy metal resistance, salinity tolerance, antibiotic resistance, EPS and biosurfactant production, and antifungal activity—offers a more holistic understanding of bacterial adaptation to polluted environments than studies that focus on single traits alone.

The strains identified, particularly *Bacillus amyloliquefaciens* Laica 1 (high Cu/Cr resistance) and *Pseudomonas putida* Laica 4 (high Cd resistance and strong antifungal activity), represent promising candidates for further investigation and potential field applications. Future work should prioritize: (i) quantitative characterization of EPS and biosurfactant production and clarification of their roles in metal sequestration; (ii) molecular identification of metal resistance and antibiotic resistance genes; (iii) in planta assays to validate biocontrol efficacy and plant growth-promoting effects under metal stress; and (iv) scale-up studies to evaluate the feasibility of using these strains for bioremediation and in agricultural systems. Addressing these points will not only confirm the mechanisms proposed here but also support the rational design of microbial consortia tailored to the remediation of multi-contaminated soils.

## 5. Conclusions

This study demonstrates that rhizospheric *Bacillus* and *Pseudomonas* strains isolated from metal-contaminated soils possess multiple, complementary traits that collectively support heavy metal tolerance and detoxification. The strains tolerated Cu, Cr, and Cd at concentrations up to 500 µg/mL and NaCl levels up to 600 mM, while also exhibiting exopolysaccharide (EPS) and biosurfactant production, antibiotic resistance, and antifungal activity. Notably, *Bacillus amyloliquefaciens* Laica 1 showed the highest resistance to copper and chromium, and *Pseudomonas putida* Laica 4 was the most cadmium-resistant strain, also displaying strong antifungal activity against *Stemphylium botryosum* (83.34 ± 2.22% inhibition). These findings highlight the potential of these strains for bioremediation and biocontrol applications.

The novel contribution of this paper to the existing literature lies in its integrative approach. While numerous studies have separately examined heavy metal resistance, salinity tolerance, antibiotic resistance, EPS production, biosurfactant synthesis, or antifungal activity in bacteria, few have explored all these traits simultaneously within the same set of strains isolated from metal-contaminated soils. This study demonstrates that these adaptive mechanisms are not isolated but coexist and potentially synergize, enabling bacteria to cope with multiple environmental stressors. By systematically characterizing these complementary traits, we provide a more holistic understanding of bacterial survival strategies in polluted ecosystems than studies focusing on single mechanisms.

The integrative approach adopted here—linking metal resistance to salinity tolerance, EPS synthesis, biosurfactant production, and antifungal activity—provides a more holistic understanding of bacterial adaptation to polluted environments than studies focusing on single traits. Microbial bioremediation offers several advantages over conventional physicochemical methods, including lower costs, reduced environmental impact, and the promotion of natural ecosystem restoration.

However, this study has limitations. Heavy metal resistance was inferred solely from optical density measurements, and associated mechanisms (EPS, biosurfactants, antibiotic resistance) were assessed through preliminary phenotypic assays. Molecular characterization of resistance genes (e.g., *czcCBA*, *cadB*, *merA*) and quantitative analyses of EPS and biosurfactant composition are needed to confirm the underlying mechanisms. Additionally, in vivo assays on infected plants are required to validate biocontrol efficacy under field conditions.

Future research should prioritize: (i) genetic and molecular investigations to identify metal resistance determinants; (ii) quantitative characterization of EPS and biosurfactants; (iii) in planta validation of plant growth-promoting and biocontrol effects; and (iv) scale-up studies for field application. Addressing these gaps will enable the rational design of microbial consortia tailored for bioremediation of multi-contaminated soils and sustainable crop protection.

## Figures and Tables

**Table 1:** Sources, geographic locations, and isolation origins of *Bacillus* and *Pseudomonas* strains.

Strain	Site	Department	GPS Localization	Plant/Soil Type
Laica 1	Tighennif	Mascara	35°24′ N 0°19′ E	*Phaseolus vulgaris* (R)
Laica 2	Tighennif	Mascara	35°24′ N 0°19′ E	*Phaseolus vulgaris* (R)
Laica 3	Tizi	Mascara	35°18′ N 0°04′ E	*Allium cepa* (R)
Laica 4	Rocade Nord	Sidi Belabbes	35°13′ N 0°37′ W	*Bulk soil* (B)

R: Rhizospheric soil; B: Bulk soil.

**Table 2:** **a.** Tukey’s multiple comparisons for copper (Cu). **b.** Tukey’s multiple comparisons for chromium (Cr). **c.** Tukey’s multiple comparisons for cadmium (Cd).

**a.**				
**Treatment**	**Summary**			
	**Laica 1**	**Laica 2**	**Laica 3**	**Laica 4**
50 µg/mL vs. 100 µg/mL	**	****	****	ns
50 µg/mL vs. 200 µg/mL	****	****	****	*
50 µg/mL vs. 400 µg/mL	****	****	****	***
50 µg/mL vs. 500 µg/mL	****	****	****	****
100 µg/mL vs. 200 µg/mL	****	****	****	ns
100 µg/mL vs. 400 µg/mL	****	****	****	***
100 µg/mL vs. 500 µg/mL	****	****	****	***
200 µg/mL vs. 400 µg/mL	****	****	****	ns
200 µg/mL vs. 500 µg/mL	****	****	****	**
400 µg/mL vs. 500 µg/mL	****	****	***	ns
ns: non-significant; *: significant difference at *p* < 0.05; **: significant difference at *p* < 0.01; ***: significant difference at *p* < 0.005; ****: significant difference at *p* < 0.001.				
**b.**				
**Treatment**	**Summary**			
	**Laica 1**	**Laica 2**	**Laica 3**	**Laica 4**
50 µg/mL vs. 100 µg/mL	****	****	****	****
50 µg/mL vs. 200 µg/mL	****	****	****	****
50 µg/mL vs. 400 µg/mL	****	****	****	****
50 µg/mL vs. 500 µg/mL	****	****	****	****
100 µg/mL vs. 200 µg/mL	****	****	****	****
100 µg/mL vs. 400 µg/mL	****	****	****	****
100 µg/mL vs. 500 µg/mL	****	****	****	****
200 µg/mL vs. 400 µg/mL	****	****	****	***
200 µg/mL vs. 500 µg/mL	****	****	****	****
400 µg/mL vs. 500 µg/mL	****	****	***	****
***: significant difference at *p* < 0.005; ****: significant difference at *p* < 0.001.				
**c.**				
**Treatment**	**Summary**			
	**Laica 1**	**Laica 2**	**Laica 3**	**Laica 4**
50 µg/mL vs. 100 µg/mL	***	***	****	****
50 µg/mL vs. 200 µg/mL	****	****	****	****
50 µg/mL vs. 400 µg/mL	****	****	****	****
50 µg/mL vs. 500 µg/mL	****	****	****	****
100 µg/mL vs. 200 µg/mL	****	****	****	****
100 µg/mL vs. 400 µg/mL	****	****	****	****
100 µg/mL vs. 500 µg/mL	****	****	****	****
200 µg/mL vs. 400 µg/mL	****	****	****	****
200 µg/mL vs. 500 µg/mL	****	****	****	****
400 µg/mL vs. 500 µg/mL	****	****	****	****
***: significant difference at *p* < 0.005; ****: significant difference at *p* < 0.001.				

**Table 3:** Tukey’s multiple comparisons for salinity tolerance.

Treatment	Summary			
	Laica 1	Laica 2	Laica 3	Laica 4
0 mM vs. 200 mM	****	ns	*	****
0 mM vs. 400 mM	****	****	****	****
0 mM vs. 600 mM	****	****	****	****
200 mM vs. 400 mM	****	****	****	****
200 mM vs. 600 mM	****	****	****	****
400 mM vs. 600 mM	****	****	****	****

ns: non-significant; *: significant difference at *p* < 0.05; ****: significant difference at *p* < 0.001).

**Table 4:** Antibiotic resistance of *Bacillus* and *Pseudomonas* strains.

Antibiotics	Codes	Concentrations (µg)	Strains			
			Laica 1	Laica 2	Laica 3	Laica 4
Imipenem	IMP	10	S	S	R	R
Amikacin	AK	30	S	S	S	S
Ampicillin	AM	10	S	S	R	R
Gentamicin	GEN	10	S	S	S	nd
Sodium fusidate	FC	10	R	S	R	R

R: resistant; S: susceptible, nd: not determined.

**Table 5:** Production of exopolysaccharides by *Bacillus* and *Pseudomonas* Strains.

Strains	Colony Aspect	Observation	EPS Production
Laica 1	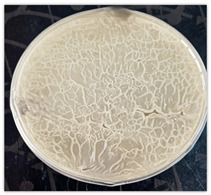	None	−
Laica 2	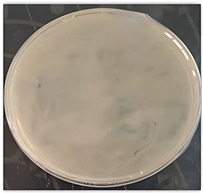	Viscous and slimy	+
Laica 3	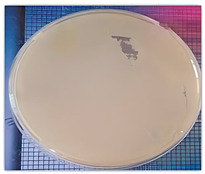	None	−
Laica 4	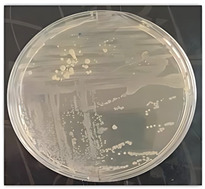	Viscous and slimy	+

EPS: exopolysaccharide; −: No production; +: production.

**Table 6:** Tukey’s multiple comparison for biosurfactant production.

Summary	Treatment
ns	Laica 1 vs. Laica 2
****	Laica 1 vs. Laica 3
*	Laica 1 vs. Laica 4
****	Laica-2 vs. Laica 3
**	Laica 2 vs. Laica 4
**	Laica 3 vs. Laica 4

ns: non-significant; *: significant difference at *p* < 0.05; **: significant difference at *p* < 0.01; ****: significant difference at *p* < 0.001.

**Table 7:** Tukey’s Multiple Comparisons for Antifungal Activity.

Treatment	Summary	
	Fop	St-bt
Laica 1 vs. Laica 2	****	**
Laica 1 vs. Laica 3	****	****
Laica 1 vs. Laica 4	****	****
Laica-2 vs. Laica 3	****	****
Laica 2 vs. Laica 4	****	****
Laica 3 vs. Laica 4	**	****

Fop: *Fusarium oxysporum* f. sp. *phaseoli*; St-bt: *Stemphylium botryosum*; **: significant difference at *p* < 0.01; ****: significant difference at *p* < 0.001.

## Data Availability

The original contributions presented in this study are included in the article. Further inquiries can be directed to the corresponding author.
